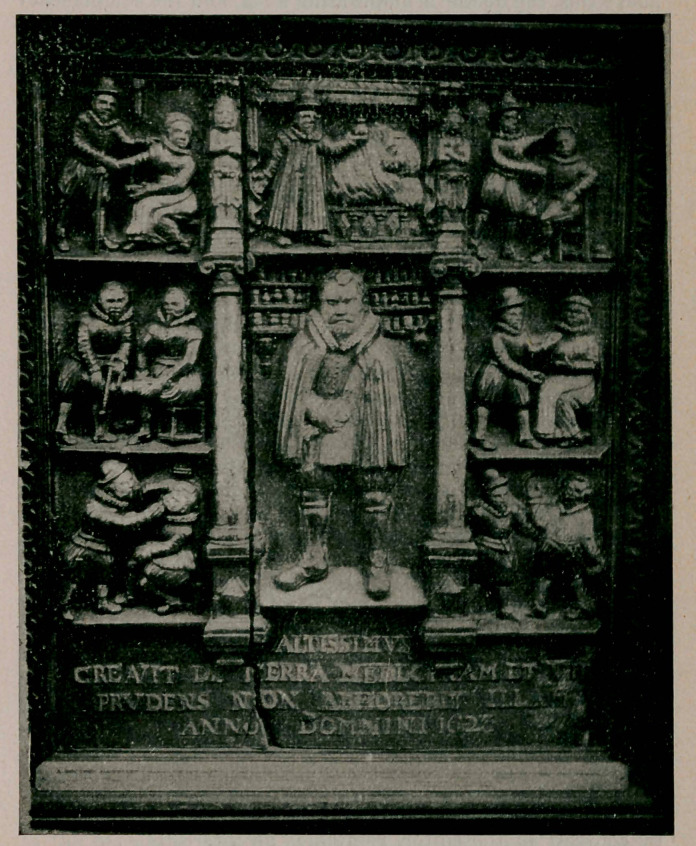# Our Contemporaries

**Published:** 1915-02

**Authors:** 


### OUR CONTEMPORARIES.

---

The Lancet-Clinic,'' under the caption of ''The Municipal Hospital and the Municipal University,'' strongly advocates the former and that it should be closely connected with a medical school. Numerous instances are cited but, so far as we are aware, there is no strictly municipal medical school, either separate or constituting part of a municipal university. New York and Philadelphia maintain strictly municipal colleges but without professional schools. So far as we can see, our contemporary has not taken a decided stand for or against municipal universities or medical schools directly under control of municipal educational departments.

### An Old English Sign Board.

The original of this sign-board is in the Royal College of Surgeons, Lincoln Inn Fields, London. The inscription on the bottom states that it was formerly the property of the late

McManly Sims, F. R. S. E., and was brought to London from Poole some years ago. It is made of material resembling plaster and is painted in dull colors, the predominant tints being brown and dark green.

The sign-board was apparently applied as a panel to the front door and quaintly advertises the doctor's activities to prospective customers. Beginning below and to the left, we observe that the dental art has not yet been divorced from the purely medical, for the doctor is extracting a tooth. We also learn that the dental forceps had not yet been invented for he is using the old-fashioned lever. In the compartment directly above this, the doctor is amputating a leg, and the complacency with which the patient undergoes this disfigurement is striking. Above this a phlebotomy is being performed. Details that may go unnoticed are the staff which the patient grasps with the hand to cause greater engorgement of the veins, and a bandage suspended from the ceiling.

In the middle compartment the doctor is dressed in his academic gown at the bedside of the patient holding a flask of urine to the light. To the right of this representation, a reduction of the shoulder is taking place. Below this the physician is examining a swelling of the breast. The lowermost compartment on the right represents a scene which is difficult to explain. Many theories have been propounded as to nature of the operation that is being performed, but the concensus of opinion seems to be that the doctor is taking his fee. This operation resembles a modern hold-up in its details. It will also be noted that the patient is already coatless, while the left hand is holding the hat away from the doctor. Whether this means that the latter has already taken the coat in part payment and that the patient is protecting his hat against a similar fate must remain a problem unsolved.

The large middle compartment represents the doctor in an attitude of serene meditation, while the vials on the shelves behind afford assurance that he can prescribe medicine as well as perform operations. The Latin inscription is scriptural and translated, reads, "The Most High has created medicine from the earth and the prudent man does not abhor them."—Eli Moschowitz, M. D. The Medical Pickwick, January, 1915.

---

The "Medical Pickwick," limited to history, humor and literature and not entering the field of Medical science or practice, has appeared. The publication office is Saranac Lake. The editor is our old college friend, Dr. Samuel Max Brickner, formerly of New York and a noted gynaecologist. We commend this new phase of medical journalism to our readers and, by courtesy of the editor, reproduce an interesting cut.

The "Vermont Medical Monthly" has discontinued publication.

---

The "Boston Medical and Surgical Journal," one of the two American medical publications older than ours, has become the official organ of the Massachusetts Medical Society.

## Figures and Tables

**Figure f1:**